# Effects of Smoking and Smoking Cessation on the Intestinal Microbiota

**DOI:** 10.3390/jcm9092963

**Published:** 2020-09-14

**Authors:** Marcus G. Sublette, Tzu-Wen L. Cross, Claudia E. Korcarz, Kristin M. Hansen, Sofia M. Murga-Garrido, Stanley L. Hazen, Zeneng Wang, Madeline K. Oguss, Federico E. Rey, James H. Stein

**Affiliations:** 1 Department of Medicine, University of Wisconsin School of Medicine and Public Health, Madison, WI 53792, USA; msublette@uwhealth.org (M.G.S.); ck4@medicine.wisc.edu (C.E.K.); khansen2@uwhealth.org (K.M.H.); mkoguss@medicine.wisc.edu (M.K.O.); 2 Department of Nutrition Science, Purdue University, West Lafayette, Indiana, 47907, USA; tlcross@purdue.edu; 3 Department of Bacteriology, University of Wisconsin, Madison, WI 53706, USA; murgagarrido@wisc.edu (S.M.M.-G.); hazens@ccf.org (S.L.H.); ferey@wisc.edu (F.E.R.); 4 Department of Cardiovascular and Metabolic Sciences, Lerner Research Institute, Cleveland Clinic, Cleveland, OH 44195, USA; wangz2@ccf.org

**Keywords:** cardiovascular disease, gut flora, microbiome, smoking, smoking cessation

## Abstract

We evaluated associations of smoking heaviness markers and the effects of smoking cessation on the intestinal microbiota and cardiovascular disease risk factors in current smokers undertaking a quit attempt. Participants were current smokers enrolled in a prospective randomized clinical trial of smoking cessation therapies with visits at baseline, 2, and 12 weeks. Genomic DNA was extracted from fecal samples followed by 16S rRNA gene sequencing and analysis using the QIIME2 software workflow. Relative abundances of bacterial taxa and alpha- and beta-diversity measures were used for comparisons. The 36 smokers were (mean (standard deviation)) 51.5 (11.1) years old (42% male) and smoked 15.1 (6.4) cigarettes per day for 22.7 (11.9) pack-years. Relative abundances of the phylum Actinobacteria correlated with pack-years (rho = −0.44, *p* = 0.008) and Cyanobacteria correlated with CO levels (rho = 0.39, *p* = 0.021). After 12 weeks, relative abundances of the phylum Bacteroidetes increased (*p*_ANCOVA_ = 0.048) and Firmicutes decreased (*p*_ANCOVA_ = 0.036) among abstainers compared to continuing smokers. Increases in alpha-diversity were associated with heart rates (rho = −0.59, *p* = 0.037), systolic blood pressures (rho = −0.58, *p* = 0.043), and C-reactive protein (rho = −0.60, *p* = 0.034). Smoking cessation led to minor changes in the intestinal microbiota. It is unclear if the proven health benefits of smoking cessation lead to salutary changes in the intestinal microbiota.

## 1. Introduction

The distal gut of humans is inhabited by dynamic and complex microbial communities that complement and influence human biology. Early studies implicated alterations in gut microbial composition and derived metabolic products in disease states such as inflammatory bowel disease, obesity, hypertension, diabetes mellitus, and rheumatologic conditions [1,2]. The gut microbiota appears to play a pathophysiologic role in atherosclerosis and cardiovascular disease, possibly through interactions with the immune system resulting in chronic systemic inflammation, by contributions to lipid metabolism, and/or through direct interactions of microbial-derived products such as trimethylamine N-oxide (TMAO) with vascular endothelium and platelets [3,4,5,6,7].

Dietary changes, antibiotics, and cigarette smoking, among several other factors, can modify the composition of the oral and gut microbiota [8,9,10,11,12]. Cigarette smoke may modulate gut microbiota by upregulating oxidative stress-related enzymes in gut immune tissue, altering the gut mucin layer, expression of intestinal tight junction proteins, and local acid/base imbalance in the colon through direct toxic effects from the myriad compounds in tobacco smoke, or through the spread of bacteria directly from cigarettes [12,13]. In some reports the fecal microbiome of smokers resembled that of patients with inflammatory bowel disease, a disease state that increases cardiovascular disease risk [12,14]. Although there is evidence that the intestinal microbiota of smokers differs from non-smokers [14,15], it is not known whether smoking cessation changes the gut microbiota and how these changes may relate to pre-quit smoking heaviness, weight gain, and cardiovascular disease risk factors. A previous report described profound changes in the intestinal microbiota following smoking cessation; however, this was a very small study, and the observed microbiome changes were not integrated with vascular and inflammatory biomarkers [12,14,15].

In this study, we sought to address this knowledge gap by evaluating associations of smoking heaviness markers and the effects of smoking cessation on the gut microbiota and microbial metabolites in current smokers undertaking a quit attempt. We also measured cardiovascular disease risk factors and non-invasive arterial parameters that reflect arterial injury and function. In contrast with the previous published work [12,14,15], our results suggest that smoking cessation is associated with minor changes in the gut microbiome.

## 2. Materials and Methods

### 2.1. Participants

The University of Wisconsin Health Sciences Institutional Review Board approved this study on 7 December 2017 (identification number 2017–1013). This study was conducted in accordance with all university, state, and federal research regulations. All participants provided free informed consent. Participants were recruited from the ongoing Quitting Using Intensive Treatment Study (QUITS) Trial (NCT03176784) and enrolled from 19 February to 16 August 2019. The main QUITS inclusion criteria required that participants be at least 18 years old; smoke ≥10 cigarettes per day; not use pipe tobacco, cigars, snuff, e-cigarettes, or chewing tobacco in the preceding 30 days; and use an acceptable birth control method. Main exclusion criteria were as follows: stage 5 or greater chronic kidney disease; hospitalization for a stroke, heart attack, congestive heart failure, or uncontrolled diabetes mellitus within the past year; or alcohol/substance dependence. This sub-study also excluded individuals with factors known to affect the gut microbiota, including the use of systemic antibiotics, corticosteroids, immunomodulators, or commercial probiotics in the last 6 months, major gastrointestinal surgery in the past 5 years, and active inflammatory bowel disease or other gastrointestinal disorders. 

### 2.2. Design

All participants received varenicline therapy (1 mg daily) and either placebo or nicotine patches (14 mg for 2 weeks pre-quit and then 10 weeks post-quit, then 7 mg patches for weeks 11 and 12 after the quit date). Smoking cessation was confirmed by 7-day self-report and verified by exhaled carbon monoxide (CO, Micro^+^™ Smokerlyzer^®^ CO monitor, Bedfont Scientific Ltd., Maidstone, Kent, England) levels. Urinary cotinine levels were not measured since participants used nicotine patches. Participants’ vital signs, smoking history, smoking heaviness markers (exhaled CO, cigarettes smoked per day), fasting laboratory tests, and stool samples were obtained at baseline (immediately prior to the quit attempt), 2 weeks post-quit attempt, and 12 weeks post-quit attempt. At each visit they completed a food-frequency questionnaire targeted at consumption and changes in consumption of foods that are rich in lecithin, choline and carnitine, as well as fat, sugar, and other nutritional components. Carotid-femoral pulse wave velocity (PWV) and brachial artery flow-mediated dilation (FMD) were measured at each visit. Carotid ultrasound to detect plaques was performed at the baseline visit.

### 2.3. Processing and Analysis of Fecal Samples

Fresh stool samples were collected by participants and kept refrigerated (4–8 °C) until delivery to the microbiology lab where an aliquot of feces was immediately stored at −80 °C until further processing. Fecal samples were kept refrigerated for an average of 3 h and 43 min between collection and storage, ranging from 40 min to 8 h and 5 min. 

#### 2.3.1. DNA Extraction

Genomic DNA was extracted from undiluted fecal aliquots using a bead-beating protocol [16]. Briefly, feces was re-suspended in a solution containing 500 μL of extraction buffer (200 mM Tris (pH 8.0), 200 mM NaCL, 20 mM EDTA), 210 μL of 20% SDS, 500 μL phenol:chloroform:isoamyl alcohol (pH 7.9, 25:24:1, Invitrogen, Carlsbad, CA), and 500 μL of 0.1 mm diameter zirconia/silica beads. Samples were mechanically disrupted using a bead beater (BioSpec Products, Barlesville, OK; maximum setting for 3 min at room temperature), followed by centrifugation, recovery of the aqueous phase, and precipitation with sodium acetate and isopropanol. QIAquick 96-well PCR Purification Kit (Qiagen, Germantown, MD, USA) was used to remove contaminants. Isolated DNA was eluted in Tris-EDTA buffer and stored at −80 °C until further use. 

#### 2.3.2. Sequencing

PCR was performed using universal primers flanking the variable 4 (V4) region of the bacterial 16S rRNA gene [16]. PCR products were purified with the QIAquick 96-well PCR Purification Kit (Qiagen, Germantown, MD, USA). Samples were quantified by Qubit Fluorometer (Invitrogen, Carlsbad, CA) and equimolar pooled. The pool was sequenced at the University of Wisconsin-Madison Biotechnology Center with the MiSeq 2x250 v2 kit (Illumina, San Diego, CA, USA). DNA extraction blanks, PCR blanks, and technical duplications for both extractions and PCRs were employed to ensure proper sample handling throughout the library preparation process. 

#### 2.3.3. Analysis

Sequences were processed using QIIME 2 pipeline (2019.1) [17]. Briefly, demultiplexed paired-end sequences were imported using Casava 1.8 format and denoised using DADA2 [18] to obtain amplicon sequence variant (ASV) table. Singletons (ASV that were observed fewer than 2 times) and ASVs that were present in less than 10% of the samples were discarded. A naive Bayes taxonomy classifier was trained on the Greengenes [19] reference database (clustered at 99% similarity). This classifier was used to assign taxonomy to ASV [20]. ASVs were then collapsed based on genus-level taxonomy. An even sampling depth (sequences per sample) of 4135 sequences per sample was used for assessing alpha- and beta-diversity measures. Pielou’s evenness index [21], Faith’s phylogenetic diversity (PD), and the Shannon diversity index were used to measure alpha-diversity. Beta-diversity was calculated using Bray–Curtis, Jaccard, and weighted and unweighted UniFrac metrics [22]. 

### 2.4. Measurements of Inflammatory Markers

Participants fasted overnight (≥8 h) and refrained from consuming fish or other seafood the day before venipuncture, after which plasma aliquots were isolated by centrifugation and frozen at −70 °C. Stable isotope dilution liquid chromatography with on-line tandem mass spectrometry (LC/MS/MS) was used for quantification of plasma TMAO as previously described [23]. Hemoglobin and white blood cell counts were measured at the University of Wisconsin Hospital and Clinics using standard lab techniques on Sysmex XE 2100 analyzers (Sysmex, Inc., Mundelein, IL, USA). High-sensitivity C-reactive protein was measured using an immunoturbidometric method on a Cobas 600 c501 analyzer (Roche Diagnostics, Inc., Indianapolis, IN, USA). Serum glucose, total cholesterol, high-density lipoprotein cholesterol, and triglycerides were measured on the same analyzer, and low-density lipoprotein cholesterol was estimated using the Friedewald formula [24].

### 2.5. Arterial Measures

Carotid-femoral PWV was determined by applanation tonometry of the right carotid and femoral artery pulsations using a SphygmoCor system (AtCor Medical, Inc., Lisle, IL, USA) as described previously. Brachial artery reactivity testing for measurement of FMD was performed using the lower-arm occlusion technique, a linear array vascular ultrasound transducer (L12-3), and a Phillips Cx50 ultrasound system as described previously [25,26]. The right and left carotid arteries were imaged using a Siemens S2000 ultrasound system with a 9L4 transducer as described previously. Carotid plaques were defined as a discrete, focal wall thickening ≥1.5 cm or focal thickening at least 50% greater than the surrounding IMT and summed to create a score of 0–12 [25,26]. 

### 2.6. Statistical Analysis

This was a pilot designed to identify plausible associations between smoking heaviness, metabolic markers, and the composition of the intestinal microbiota and their changes 12 weeks after a quit attempt. All arterial measures were exploratory. All analyses were performed using XLStat software (Addinsoft, France) in R (R Core Team, 2014). All parameters were described by means and standard deviations. Distributions were inspected for frequent zero values and non-linearity. At baseline, Spearman’s rank correlations were used to compare relative abundances of microbial taxonomy, smoking heaviness markers, cardiovascular disease risk factors, and arterial function measures. We confirmed all correlations by inspecting the scatterplots of the distributions for outliers and zero values. ANCOVA was used to compare temporal changes in intestinal microbiota abundances and diversity measures by eventual cessation group (abstainers vs. continuing smokers) and each baseline measure. Baseline and week 12 data are presented. Given the exploratory nature of our study, we estimated statistical power using the genus taxonomic level with up to 13 different categories. With a sample of size of 30 we would have 80% power to detect a rank correlation of 0.57 between the abundance metrics and outcome measures using a two-sided test for significance at a taxonomic family-wise 0.05 level; however, we did not perform formal adjustments for multiple comparisons. We anticipated that 50% of our participants would quit smoking by the 12-week visit. 

## 3. Results

### 3.1. Participant Characteristics 

A total of 36 smokers attended the baseline visit. They were 51.5 (11.1) years old, 58% were female, and they smoked 15.1 (6.4) cigarettes per day for 22.7 (11.9) pack-years. Their exhaled CO levels were 17.6 (9.3) ppm (Table 1). Relative abundances of bacteria at the phylum level, as well as alpha- and beta-diversity measures, are presented in Table 2. Greengenes classifier assigned usable raw reads to 11 phyla, 35 families, and 55 genera. The most abundant phyla included Firmicutes (66.9% of sequences), Bacteroidetes (20.3% of sequences), and Proteobacteria (10.3% of sequences).

### 3.2. Baseline Correlations between Bacterial Taxa Abundances, TMAO, Microbial Diversity Scores, and Smoking Measures

At baseline, pack-years of cigarettes smoked was correlated inversely with the relative abundance of Actinobacteria (rho = −0.44, *p* = 0.008), and exhaled CO correlated positively with the relative abundance of Cyanobacteria (rho = 0.39, *p* = 0.021). No significant associations between Actinobacteria or Cyanobacteria and smoking measures (pack-years, cigarettes smoked/day, exhaled CO) were identified, nor were taxa at the family or genus levels within these two phyla. The relative abundance of the Actinobacteria phylum was inversely correlated with age (rho = −0.44, *p* = 0.007) and carotid artery plaque score (rho = −0.33, *p* = 0.049) and directly correlated with fasting glucose (rho = 0.038, *p* = 0.021). Actinobacteria and Cyanobacteria were not associated with TMAO levels, hsCRP levels, or any other cardiovascular disease risk factors or arterial measures. TMAO levels did not have significant correlations with any of the cardiovascular disease measures (carotid plaque score, PWV, brachial FMD; data not shown). Exhaled CO levels were associated with the Firmicutes genera *Dorea* (rho = 0.43 *p* = 0.009) and *Streptococcus* (rho= −0.47, *p* = 0.022), otherwise none of the smoking heaviness measures was significantly associated with other diversity indexes or bacterial abundances at the phyla, family, or genera levels. 

### 3.3. Effects of Smoking Cessation and Continued Smoking on Microbiota Abundances, Diversity Scores after 2 and 12 weeks

Of the 29 participants that returned for the week 2 visit, 17 had successfully quit smoking and 12 continued to smoke. We did not observe any significant between-group differences in relative abundances of bacteria or diversity measures at this early time point. 

**Figure 1:**
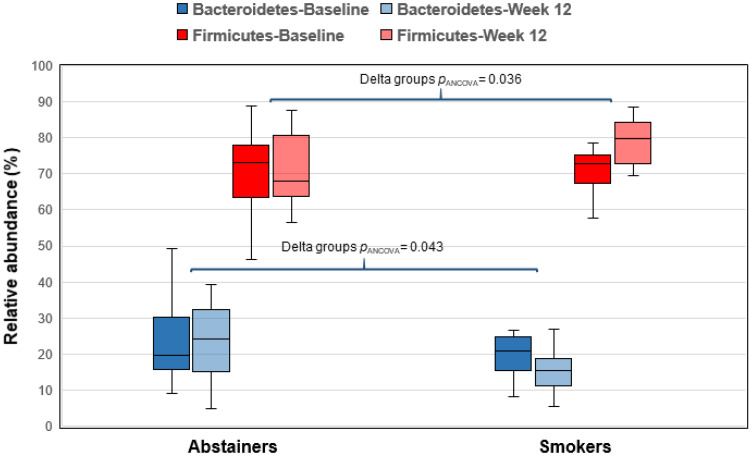
Changes in relative abundances of Bacteroidetes and Firmicutes from baseline to week 12 by smoking status.

Of the 26 participants that returned for the week 12 visit, 14 successfully quit smoking and 12 continued to smoke. After 12 weeks, those who quit smoking had greater reductions in levels of exhaled CO than continuing smokers (−14.0 (10.4) vs. +2.1 (7.6) ppm, group effect *p*_ANCOVA_ < 0.001). Significant between-group differences in changes for the 12 week visit were not observed for cardiovascular disease risk factors, hsCRP, TMAO, PWV, and brachial artery FMD (data not shown). Significant between-group differences were not observed for relative abundances of Actinobacteria (*p*_ANCOVA_ = 0.150) or Cyanobacteria (*p*_ANCOVA_ = 0.165). However, relative abundances of Bacteroidetes increased (*p*_ANCOVA_ = 0.048) and Firmicutes decreased (*p*_ANCOVA_ = 0.036) among successful abstainers compared to continuing smokers (Table 3, Figure 1). Among the Firmicutes, we identified a small but statistically significant decrease in the genus *Ruminococcus* (*p*_ANCOVA_ = 0.027) and a small increase in an undefined genus within the order Closteridiales (*p* = 0.043). No other significant changes between groups were identified for relative abundances of bacteria at the phylum level. 

Although changes in alpha- and beta-diversity measures over time did not differ between successful abstainers and continuing smokers, increases in alpha-diversity 12 weeks following a smoking cessation attempt were associated with lower heart rates (rho = −0.59, *p* = 0.037), systolic blood pressures (rho = −0.58, *p* = 0.043), and C-reactive protein levels (rho = −0.60, *p* = 0.034).

At week 12, there were no significant differences in the gut microbiota between participants that received a placebo or a nicotine patch. As expected, the nicotine patch users were more successful at quitting with significantly greater reductions in CO levels at the week 12 visit (*p* = 0.006). Dietary changes in meat, poultry, fish or shellfish, eggs, diary, fatty foods, sugary foods, fiber, and overall amount of daily food consumption were not different between successful abstainers and continuing smokers (data not shown). Among abstainers, changes in TMAO levels were associated with an increase in sugary foods intake (*r* = 0.57, *p* = 0.045) but not higher plasma levels of glucose. 

## 4. Discussion

In this study of daily cigarette smokers, pack-years of smoking were inversely correlated with relative abundances of Actinobacteria, and exhaled CO levels were inversely correlated with the abundance of Cyanobacteria. The number of cigarettes smoked per day was not associated with any bacterial taxa at the phylum level. Relative abundances of these bacteria were not associated with plasma TMAO, cardiovascular disease risk factors, or arterial measures. After a quit attempt, successful abstainers had significantly lower exhaled CO levels, but we did not observe significant changes in TMAO levels. We also did not observe between-group differences in relative abundances of Actinobacteria or Cyanobacteria. Our primary observation was that relative abundances of Bacteroidetes increased and Firmicutes decreased in successful abstainers; however, we observed the reverse pattern in the continued smoker group, such that between-group differences in changes in the abundances of these bacteria were statistically significant. We also did not observe significant changes in alpha- or beta-diversity with smoking cessation. Overall, we observed relatively minor effects of smoking cessation on the microbiome. 

An increased Bacteroidetes:Firmicutes ratio has been observed in individuals with obesity, type I diabetes mellitus, rheumatoid arthritis, and systemic lupus erythematosus. All of these disease states carry an increased risk of atherosclerotic disease [12,15,27,28,29,30,31,32]. It has been hypothesized that increases in Bacteroidetes lead to production of lipopolysaccharide which upregulates inflammatory pathways [33]; however, some strains of Bacteroidetes produce non-inflammatory lipooligosaccharide rather than lipopolysaccharide [34]. Bacteroidetes also are important producers of acetate in the gut, which is readily absorbed and can be used for cholesterol production [1,27]. 

Very few studies have examined the fecal microbiome of smokers after cessation. An observational study of 10 smokers suggested smoking cessation led to a decrease in the Bacteroidetes:Firmicutes ratio [15]. Our prospect study of three times as many active smokers did not confirm their findings, but it was consistent with a cross-sectional, population-based study which showed an increased Bacteroidetes:Firmicutes ratio in the fecal microbiomes of current smokers compared to never smokers and former smokers [35].

Decreases in fecal bacterial diversity have been linked to obesity, and in a small study, smoking cessation was associated with a short-term increase in alpha-diversity [15]. We did not observe significant changes in alpha- or beta-diversity with smoking cessation, though we observed increases in alpha-diversity over time were associated with improvement in cardiovascular disease risk factors in abstainers. After a quit attempt, higher Pielou’s evenness index was associated with lower heart rate, and higher Shannon diversity index was associated with lower systolic blood pressure and hsCRP. 

### Study Limitations

This study has some inherent limitations including a relatively small sample size, though to our knowledge this is the largest study of smoking and cessation on the fecal microbiota to date. Follow-up was only 12 weeks, and several subjects dropped out of the study, as is very common in studies of smoking cessation pharmacotherapy, so we cannot exclude a more long-term effect of smoking cessation [25,36,37]. This study was limited to current smokers making a quit attempt, so we cannot compare our findings to non-smokers. The gut microbiota is complex and susceptible to many other factors that are difficult to control in free-living individuals with inherent genetic variations. Because of this variation and the small sample size, *p*-values derived from our analyses, even when statistically significant (i.e., *p* < 0.05), no longer achieve this threshold after correction for multiple hypotheses. Our findings need to be further validated in a larger study. 

## 5. Conclusions

Smoking cessation has minor effects on the composition of the gut microbiome. Among current smokers, relative abundances of the phylum Actinobacteria were inversely associated with pack-years of smoking, and abundance of the phylum Cyanobacteria were directly associated with CO levels. In successful abstainers, relative abundances of the phyla Bacteroidetes increased and Firmicutes decreased, a pattern of uncertain clinical significance. We did not observe significant changes in alpha- or beta-diversity with smoking cessation. It is unclear if the proven health benefits of smoking cessation lead to salutary changes in the intestinal microbiota and if such changes affect cardiovascular disease risk.

## Figures and Tables

**Table 1:** Participant characteristics at baseline (*n* = 36).

	Mean	Standard Deviation	Range
Age (years)	51.4	11.2	26–71
Male/Females (%)	42.0/58.0	-	-
Cigarettes/day	15.1	6.4	5.1–35.0
Pack-years	22.7	12.0	2.5–52.0
Exhale Carbon monoxide (ppm)	17.6	9.3	5–41
Body-mass index (kg/m^2^)	29.6	8.4	20.7–67.9
Heart rate (bpm)	72.3	10.7	57–100
Systolic blood pressure (mmHg)	131.4	16.5	102–162
Diastolic blood pressure (mmHg)	77.6	9.0	66–95
Non-high-density lipoprotein cholesterol	160.3	52.6	69–274
Glucose (mg/dL)	92.4	9.7	70–109
C-reactive protein (mg/L)	4.6	7.5	0.1–39.5
Trimethylamine N-oxide (microM)	3.75	2.11	1.11–13.18
White blood cell count (K/μL)	8.4	2.2	4.6–13.9
Carotid-femoral pulse wave velocity (m/s)	7.46	1.84	4.80–12.95
Brachial artery flow-mediated dilation (%)	4.57	2.91	0.55–12.05
Carotid plaque score (0–12)	3.0	2.8	0–10

**Table 2:** Baseline relative abundances of microbiota and diversity measures.

	Mean	Standard Deviation	Range
Microbiota Relative Abundances (%)			
Actinobacteria	4.17	4.37	0.0–15.90
Cyanobacteria	0.04	0.13	0.0–0.72
Verrucomicrobia	0.70	2.55	0.0–14.41
Euryarchaeota	0.10	0.24	0.0–0.96
Bacteroidetes	23.40	10.5	8.10–49.30
Firmicutes	70.20	10.0	46.10–88.70
Fusobacteria	0.011	0.44	0.0–2.52
Proteobacteria	1.26	1.02	0.0–5.70
Alpha-Diversity Measures (units)			
Pielou’s evenness	0.77	0.06	0.58–0.84
Faith PD	8.32	1.68	4.78–12.11
Shannon	5.14	0.60	3.05–6.12

**Table 3:** Effect of quit attempt on changes in bacterial abundance and diversity measures from baseline to week 12.

	Beta	Standard Error	*p*-Value
Microbiota Relative Abundances (%)			
Actinobacteria	1.2	0.8	0.15
Cyanobacteria	0.0	0.0	0.17
Verrucomicrobia	0.0	0.1	0.92
Euryarchaeota	0.0	0.4	0.99
Bacteroidetes	7.4	3.5	0.048
Firmicutes	−7.6	3.4	0.036
Fusobacteria	−0.1	0.1	0.53
Proteobacteria	0.3	0.6	0.67
Alpha-Diversity Measures (units)			
Pielou’s evenness	−0.01	0.02	0.61
Faith PD	−0.10	0.85	0.91
Shannon	−0.13	0.23	0.59
